# Genome-Wide Associations for Water-Soluble Carbohydrate Concentration and Relative Maturity in Wheat Using SNP and DArT Marker Arrays

**DOI:** 10.1534/g3.117.039842

**Published:** 2017-06-27

**Authors:** Ben Ovenden, Andrew Milgate, Len J. Wade, Greg J. Rebetzke, James B. Holland

**Affiliations:** * New South Wales Department of Primary Industries, Yanco Agricultural Institute, New South Wales 2703, Australia; † New South Wales Department of Primary Industries, Wagga Wagga Agricultural Institute, New South Wales 2650, Australia; ‡ Charles Sturt University, Graham Centre, Wagga Wagga, New South Wales 2678, Australia; § Commonwealth Scientific and Industrial Research Organisation Agriculture and Food, Canberra, Australian Capital Territory 2601, Australia; ** Plant Science Research Unit, United States Department of Agriculture-Agricultural Research Service, North Carolina State University, Raleigh, North Carolina 27695-7620; †† Department of Crop and Soil Sciences, North Carolina State University, Raleigh, North Carolina 27695-7620

**Keywords:** water-soluble carbohydrates, nonstructural carbohydrates, association analysis, genotype-by-environment interaction, molecular marker

## Abstract

Improving water-use efficiency by incorporating drought avoidance traits into new wheat varieties is an important objective for wheat breeding in water-limited environments. This study uses genome wide association studies (GWAS) to identify candidate loci for water-soluble carbohydrate accumulation—an important drought-avoidance characteristic in wheat. Phenotypes from a multi-environment trial with experiments differing in water availability and separate single nucleotide polymorphism (SNP) and diversity arrays technology (DArT) marker sets were used to perform the analyses. Significant associations for water-soluble carbohydrate accumulation were identified on chromosomes 1A, 1B, 1D, 2D, and 4A. Notably, these loci did not collocate with the major loci identified for relative maturity. Loci on chromosome 1D collocated with markers previously associated with the high molecular weight glutenin *Glu-D1* locus. Genetic × environmental interactions impacted the results strongly, with significant associations for carbohydrate accumulation identified only in the water-deficit experiments. The markers associated with carbohydrate accumulation may be useful for marker-assisted selection of drought tolerance in wheat.

Reduction in grain yield and quality due to drought decrease the sustainability of farming systems, and threatens global food security (Ray *et al.* 2012; Reynolds *et al.* 2016). Incorporating traits that improve water-use efficiency (WUE) in water-limited environments into elite breeding germplasm is an important aim for wheat genetic improvement (Rebetzke *et al.* 2009; Reynolds *et al.* 2015). Water soluble carbohydrate (WSC) accumulation and remobilization are promising traits that could contribute to improved grain-filling under water-limited conditions, and, consequently, improved WUE (Bidinger *et al.* 1977; Pheloung and Siddique 1991; Gebbing and Schnyder 1999; Foulkes *et al.* 2007; Piaskowski *et al.* 2016). Carbohydrate accumulation occurs when the crop synthesizes assimilate at a rate greater than sink requirement. In wheat, most of the carbohydrate is stored in the form of fructans, with a minor component of sucrose and hexose (Schnyder 1993; Wardlaw and Willenbrink 1994). Both the accumulation and remobilization of WSC is modified by environmental conditions that alter the balance between sources and sinks of assimilate. In particular, the availability of source carbon (as sucrose) affects accumulation (Xue *et al.* 2013). The WSC can be remobilized for use in growth or respiration (Kiniry 1993). However, the main sink for remobilization is the developing grain (Schnyder 1993; van Herwaarden *et al.* 1998; Takahashi *et al.* 2001), with remobilized WSC contributing as much as 30–50% of grain yield under terminal drought conditions, and 10–20% under well-watered conditions (Bidinger *et al.* 1977; Pheloung and Siddique 1991; Schnyder 1993; Gebbing and Schnyder 1999; Piaskowski *et al.* 2016).

Flowering time is a key trait associated with WSC accumulation owing to the nature of WSC accumulation across crop growth stages (Passioura 1996; Rebetzke *et al.* 2008). Accumulation of WSC increases from before anthesis to a peak at 7–20 d after anthesis (Gebbing 2003; Ehdaie *et al.* 2008; Zhu *et al.* 2010) where WSC concentration (WSCC) can reach as much as 40% of total stem weight (Schnyder 1993). After anthesis, WSC levels decline due to remobilization to other sinks. Under water deficit conditions, this peak can sometimes occur before anthesis (Goggin and Setter 2004), and remobilization is earlier and proportionally greater (Bidinger *et al.* 1977; Virgona and Barlow 1991).

A number of studies have reported on genetic control and quantitative trait loci (QTL) for WSC accumulation and related characters in wheat (Snape *et al.* 2007; Yang *et al.* 2007; Rebetzke *et al.* 2008; McIntyre *et al.* 2010; Pinto *et al.* 2010; Bennett *et al.* 2012). Mapping populations typically varied for the major developmental genes for photoperiod sensitivity and reduced plant height, which can indirectly cause much of the observed phenotypic variability for grain yield and other traits (Rattey *et al.* 2009; Bennett *et al.* 2012; Edae *et al.* 2014). The biparental populations assessed in Rebetzke *et al.* (2008) were segregating for the photoperiod sensitivity locus *Ppd-D1* and the semi-dwarfing loci *Rht-B1* and *Rht-D1*, and these loci collocated with QTL for WSCC, WSC total amount per square meter, and WSC per tiller.

Genomic strategies show significant promise for the improvement and understanding of drought tolerance traits (Langridge and Reynolds 2015). The primary objectives of this study were to identify markers associated with WSCC by genome-wide association studies (GWAS) and characterize the dependency of marker associations on environments. We conducted GWAS separately for two molecular marker sets (SNP and DArT markers) and for each experiment to assess the variability of marker-trait associations due to genotype × environment (G × E) interaction (Oldmeadow *et al.* 2011; Zila *et al.* 2013). We also conducted GWAS for relative maturity to ascertain if loci with significant associations with WSCC were due to the indirect effects of relative maturity on WSCC.

## Materials and Methods

### Genotypes used in this study

The genotypes for the GWAS analyses were selected from evaluation trials conducted in multiple environments in 2009 and 2010. Each field trial contained 990 genotypes. Some genotypes were not repeated at every experiment, with a total of 1314 genotypes tested. Thus, for relative maturity GWAS, all 990 genotypes were used in 2009 experiments, and 972 genotypes were used in 2010 experiments. The accumulation of WSC varies with plant development (Ehdaie *et al.* 2008), so the subset used for WSCC GWAS consisted of 312 breeding lines from the 2009 experiments constrained to a 3–5 d difference in anthesis date as well as 46 commercially grown varieties. For the second year of this study in 2010, except for 11 breeding lines excluded from the overall experiments, the same breeding lines and varieties were evaluated for WSCC.

### Experimental design and site locations

Experiments with contrasting irrigation and rainfed treatments were grown at Yanco Agricultural Institute (Yanco, Australia) and Coleambally Community Experimental Demonstration Farm (Coleambally, Australia) in 2009 and 2010. A split-plot design was used, in which the main-plot factor was irrigation treatment (irrigated or rainfed), and the 990 genotype entries (including the subset of genotypes for WSC measurement) were the subplot factor. There were two replicates of each treatment at each location. Genotype placement was optimized with the spatial design package DiGGer (Coombes 2002). For the laboratory phase measuring WSC using near-infrared spectroscopy (NIRS), an experimental design structured by day of measurement and NIRS instrument carousel and well was implemented to account for extraneous variation originating from laboratory processes. Samples from both field locations were pooled into one laboratory phase experimental design for each year, and the placement of genotypes within the laboratory experimental phase was also optimized with DiGGer (Coombes 2002), with partial replication of 20% of experiment field plots sampled (*i.e.*, a replication level of 1.2), following the methods in Cullis *et al.* (2006) and Smith *et al.* (2006).

All experiments were sown on a full soil profile of moisture, achieved by flood irrigating each site 4–6 wk before sowing, so that the focus on water deficit conditions would be in the later stages of crop growth. Sowing dates were targeted for the first 2 wk of May, and sowing rates were 115 kg ha^−1^ in the irrigated and 70 kg ha^−1^ in the rainfed treatments, respectively. Presowing nitrogen was targeted to be 120 kg N ha^−1^ from the combination of deep soil nitrogen (following soil testing—data not shown) and fertilizer applied at sowing. Irrigated experiments were fertilized supplementally through the growing season to a total of ∼300 kg N ha^−1^ consistent with predicted N demand by the crop. Experiments were subject to a strict weed, pest, and disease control regime to maximize yield potential. Soil moisture at each experiment was monitored using gypsum block AM400 soil moisture data loggers (Hansen, Wenatchee, WA). Onsite weather stations (Davis Instruments, Hayward, CA) were used to record rainfall and air temperature. Irrigation treatments were flood irrigated when soil water potential fell below −75 kPa. Both sites had below average rainfall and above average temperatures in 2009, while conditions at both sites in 2010 had higher rainfall and lower temperatures than average.

### Measurements and observations

Relative maturity at a common date around the median flowering date for all entries within each experiment was determined using the Zadoks decimal score for plant development (Zadoks *et al.* 1974). Scores for each field experiment were taken when most lines were in the range Z50–Z69 (head emergence to completion of anthesis).

Lines selected for WSCC analysis were sampled from a 50-cm long section of row (0.09 m^2^) when the irrigated treatments at each site were ∼180° d postanthesis, following the sampling method of Rebetzke *et al.* (2008). For WSC analysis, ∼5–10 stalks (including leaves, leaf sheaths, and heads, but not senesced plant material) were subsampled from each biomass sample, and ground to pass through a 2 mm-sized screen. Ground biomass samples were homogenized, desiccated, and scanned by NIRS with a Bruker Multi-purpose Analyzer (Bruker Optik GmbH, Ettlingen, Germany) and OPUS software (version 5.1). Scanned spectra were transformed using the first derivative and multiplicative scatter correction. Calibrations to obtain predicted WSCC values from spectra measurements were constructed using the “Quant 2 Method” component of the OPUS software with a randomly selected 10% subset of samples. WSCC for the calibration samples was determined using the alkaline ferricyanide method (Piltz and Law 2007).

### Statistical methods for phenotype values

A multiplicative mixed linear model was used to analyze the multi-experiment phenotype data for both traits following Gilmour *et al.* (1997) and Beeck *et al.* (2010). The linear mixed model is given by

$$y=X\text{τ}+Z_{g}g+Z_{u}u+\text{η}$$

where *y* is the (n×1) data vector of the response variable across *p* experiments with *N* plots per experiment; τ is a (t×1) vector of fixed effects (including linear trends across range and row) with associated design matrix X. The term u is a random component with associated design matrix $Z_{u}$and contains experimental blocking structures used to capture extraneous variation (including field range and row for both traits, and laboratory day of measurement, NIRS carousel and well for WSCC only).

The residual error is $\text{η}=\left({\text{η}}_{1},\ldots ,{\text{η}}_{p}\right),$ which, at the *j*th experiment, was assumed to have distribution ${\text{η}}_{j}∼N\left(0,{\text{σ}}_{j}^{2}R_{j}\right),$ where ${\text{σ}}_{j}^{2}$ is the residual variance for the *j*th experiment and $R_{j}$ is a matrix that contains a parameterization for a separable autoregressive AR1⊗AR1 process to model potential spatial correlation of the observations for the relative maturity analysis. For WSCC analysis, unique residual variances for each year were modeled.

The term g is a random component with associated design matrix $Z_{g}$used to model the genotype within experiment effects, which combine the genotype and G × E interaction effects. Organizing the genotype within environment effects as a matrix of rows corresponding to genotypes and columns corresponding to environments facilitates modeling g as a multiplicative *k*-factor analytic (FA) model (Smith *et al.* 2001):

$$g=\left(\text{Λ}\;⊗\;I_{m}\right)f+\text{δ}$$

where Λ is a matrix with *j*th column containing the *j*th factor loadings for the *p* experiments, f is a vector of genotype scores across the *p* experiments, and $\text{δ}=\left({\text{δ}}_{1},\ldots ,{\text{δ}}_{p}\right)$ is a residual genetic term, where, at the *j*th experiment, ${\text{δ}}_{j}∼N\left(0,{\text{σ}}_{gj}^{2}I_{m}\right),$ and ${\text{σ}}_{gj}^{2}$ is the residual genetic variance for the *j*th experiment. The term $I_{m}$ represents an m×m identity matrix.

The variance model for the combined genotype and G × E effects is given by

$$var\left(g\right)=\left(\Lambda \Lambda^{\prime }+\text{ψ}\right)⊗I_{m}\;$$

where ψ is a diagonal matrix of the *p* environment specific variances.

For each analysis, the most parsimonious FA model was identified using the Akaike Information Criterion (AIC) (Akaike 1974). The nongenetic random effects were maintained in the model if they were significant according to log likelihood ratio tests relative to the full model with all nongenetic random effects (Stram and Lee 1994). Fixed effects were tested for significance using Wald F-statistics (Kenward and Roger 1997).

Empirical best linear unbiased predictors (E-BLUPs) for phenotypic values were obtained from the FA models for each individual experiment (Kelly *et al.* 2007; Cullis *et al.* 2010). For both relative maturity and WSCC, experiments were clustered using the matrix of genetic correlations between experiments (Cullis *et al.* 2010). All data were analyzed using the software package ASReml-R (Butler *et al.* 2009), in the R statistical software environment (R Development Core Team 2012).

### Genotyping methods

Two separate marker sets were used: 985 lines from the overall experiment were genotyped using the Illumina 9k Infinum iSelect beadchip array (Cavanagh *et al.* 2013), resulting in 4883 polymorphic SNPs across the population. Similarly, 955 lines were genotyped with Diversity Arrays technology (DArT) (Akbari *et al.* 2006) resulting in 2013 polymorphic markers across the population. Genotyping included all 358 lines phenotyped for WSCC. Genotype information for SNP and DArT marker datasets were prepared separately for analysis using the R software package Synbreed (Wimmer *et al.* 2012). Imputation of missing values (3.5% for SNPs and 15% for DArTs) was performed using the software package Beagle (Browning and Browning 2009). Each marker dataset was filtered for duplicated and monomorphic markers, as well as markers with minor allele frequency of <5%. The resulting 4162 SNP markers and 1773 DArT markers were used to compute a separate scaled identity by descent relationship matrix (K) after Endelman and Jannink (2012) for each marker dataset.

Consensus maps were used for marker physical positions. For the DArT dataset this study used the Wheat Interpolated Maps (version 6) as a reference to locate the positions of DArT markers (Dr Andrzej Kilian, Diversity Arrays Pty Limited, personal communication), and for the SNP dataset the 9K Consensus Map (version 4) was used (Dr Matthew Hayden, DEPI Victoria, personal communication.).

### Linkage disequilibrium analysis

Patterns of linkage disequilibrium (LD) in the SNP and DArT marker sets were estimated using the methods of Breseghello and Sorrells (2006). Pairwise LD estimates (*r*^2^) were calculated with the software package PLINK (Purcell *et al.* 2007) for unlinked loci pairs and for syntenic loci separately. Syntenic *r*^2^ was plotted against pairwise genetic distance from the consensus maps for all chromosomes on each genome with a second degree locally weighted polynomial regression (LOESS) curve fitted to each scatter plot (Cleveland 1979). All of the unlinked *r*^2^ estimates were square-root-transformed to approximate a normal distribution, and the 5% quantile of that distribution was determined following Breseghello and Sorrells (2006). The intersection of the LOESS curve and the 5% quantile for unlinked marker pairs was taken as an estimate of the extent of LD decay within each genome following Laidò *et al.* (2014).

### GWAS methods

Separate association analyses for each trait at each experiment were performed using the phenotype E-BLUPs described above. Associations using SNP and DArT marker sets were performed separately. The compressed mixed linear model approach (Zhang *et al.* 2010) was implemented in the R software package Genome Association and Prediction Integrated Tool (GAPIT) (Lipka *et al.* 2012) as follows:

$$\hat{y}=X\text{β}+Z_{g}u+\text{η}$$

where $\hat{y}$ is the vector of E-BLUPs for one trait measured in one experiment, β is a vector of fixed effects for the corresponding design matrix (X), including a molecular marker. The vector of overall genetic line effects u (with associated design matrix $Z_{g}$) is modeled as $Var\left(u\right)=K{\text{σ}}_{a}^{2},$ where K is the relationship matrix and ${\text{σ}}_{a}^{2}$ is the estimated additive genetic variance. η is the vector of random residuals. False discovery rates (FDR) were estimated separately for each experiment following Benjamini and Hochberg (1995) with a nominal threshold of 10% to declare significant associations.

### Data availability

Supplemental Material, File S1 contains a detailed description of all Supplemental files. File S2 contains phenotype information for WSCC. File S3 contains phenotype information for relative maturity at flowering time. File S4 contains SNP genotypes for each individual. File S5 contains DArT genotypes for each individual.

## Results

### Genotype × environment interactions

Consistent with experimental weather conditions, genetic correlations for WSCC between experiments showed two distinct environment groups, with the Yanco and Coleambally 2009 rainfed experiments, which experienced terminal water deficit, forming one cluster, and the other experiments collectively representing a well-watered cluster. Within these clusters, the genetic correlations were maintained at *r*_G_ = 0.87 between the two rainfed experiments that make up the water deficit environment cluster, and ranged from *r*_G_ = 0.74–0.98 in the well-watered environment cluster. Between the two clusters, genetic correlations ranged from *r*_G_ = 0.02–0.35. For relative maturity, no environmental clustering was evident, and genetic correlations between all experiments were very high, ranging from *r*_G_ = 0.92 to *r*_G_ = 0.99.

### LD and minor allele frequency

**Figure 1:**
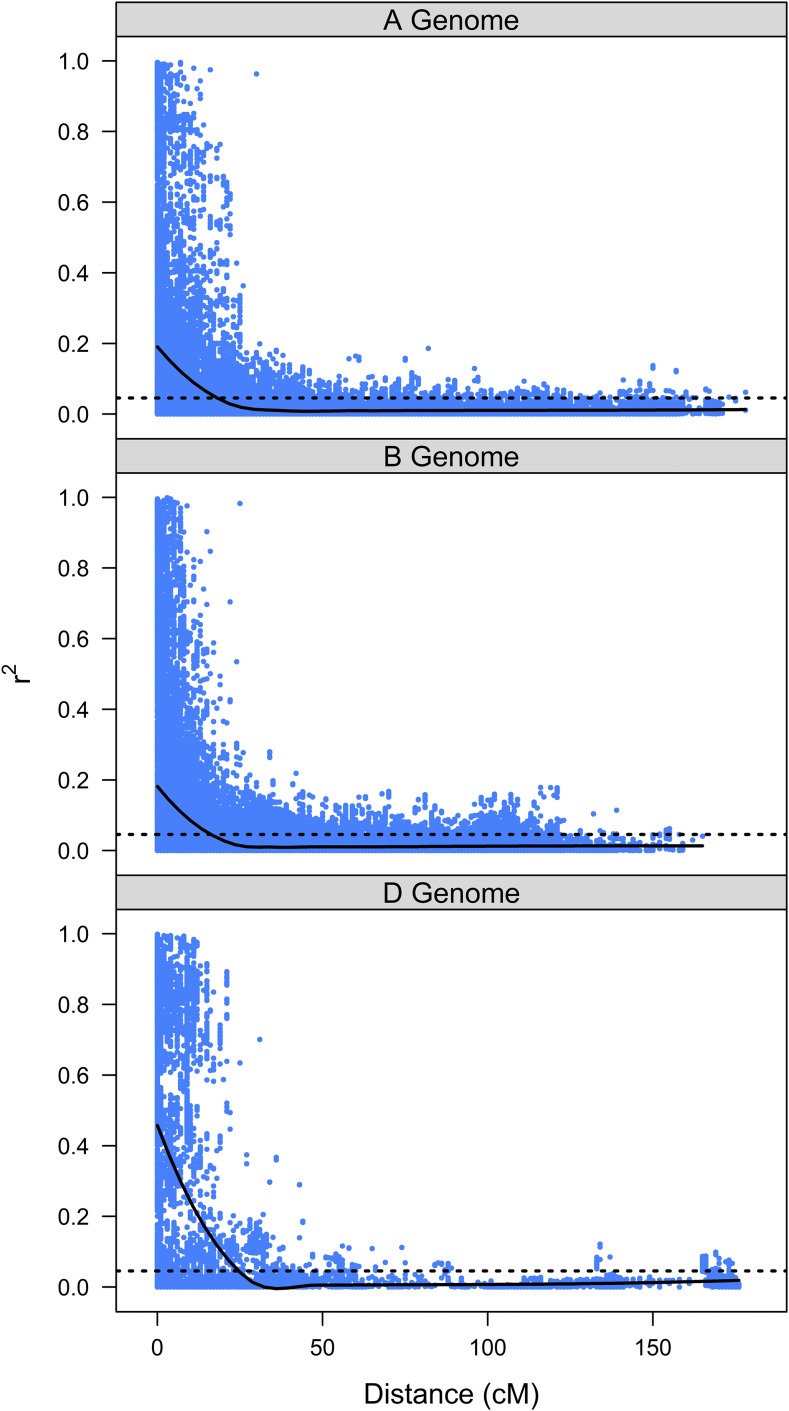
Pairwise LD estimates (*r*^2^) plotted against Euclidian pairwise marker distances for markers on the same consensus chromosome for the DArT marker set.

**Figure 2:**
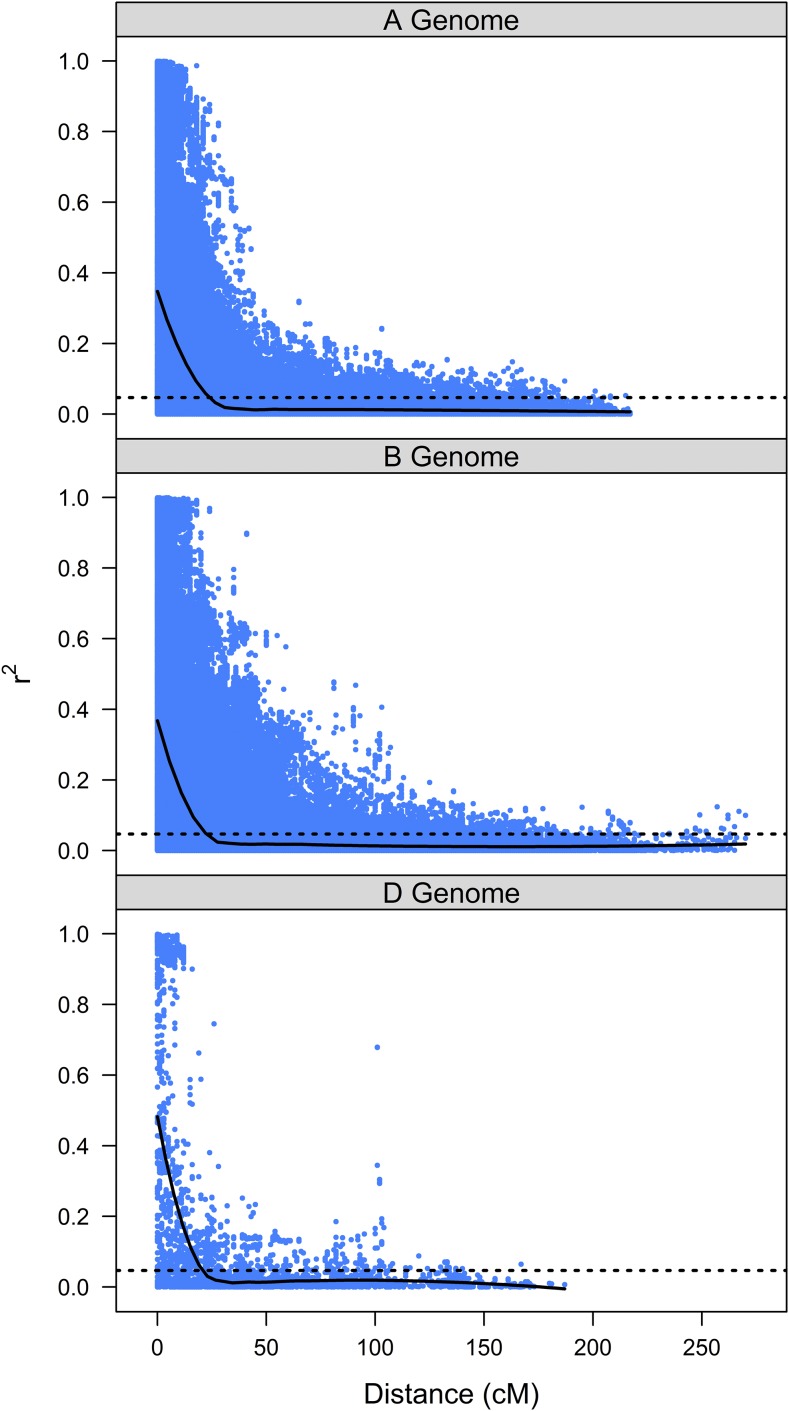
Pairwise LD estimates (*r*^2^) plotted against Euclidian pairwise marker distances for markers on the same consensus chromosome for the SNP marker set.

We evaluated the distribution of LD within chromosomes separately for each marker set and for each of the three wheat genomes. LD was more extensive with respect to linkage distances within the D genome for the DArT marker set, as the average DArT marker LD did not decrease below the 5% quantile for unlinked marker pairs (*r*^2^ = 0.0456) until the distance between markers was ≥25 cM (Figure 1). By comparison, the average LD was <0.0456 at distances of 16–18 cM for the A and B genomes respectively. In contrast, LD decreased below the 5% quantile (*r*^2^ = 0.0470) for the SNP marker set at 21 cM for both the D and B genomes, while LD for the A genome was higher at 24 cM (Figure 2).

**Figure 3:**
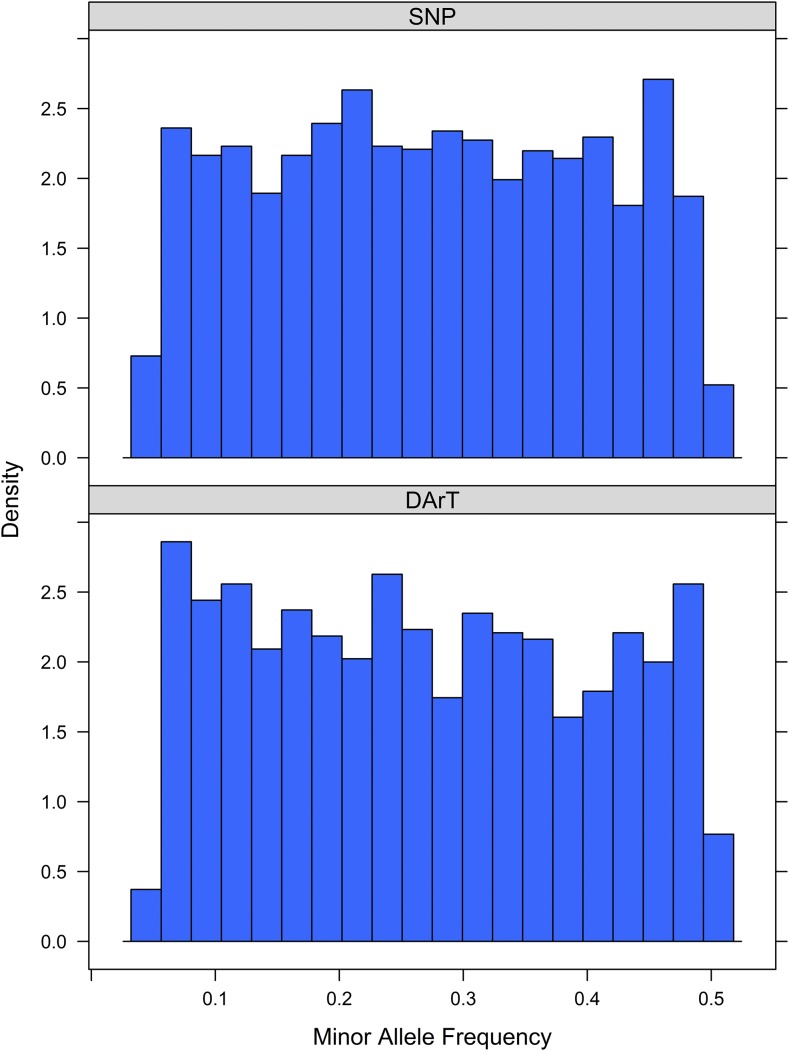
Minor allele frequency distribution for SNP and DArT marker datasets used for associations.

The minor allele frequency distribution for SNP markers was similar to that for the DArT markers, although the DArT markers had a lower proportion of the rarest allele class (MAF = 5–7.25%; Figure 3). SNPs with MAF below 5% were not included in the analysis because of their reduced power for GWAS.

### GWAS

**Figure 4:**
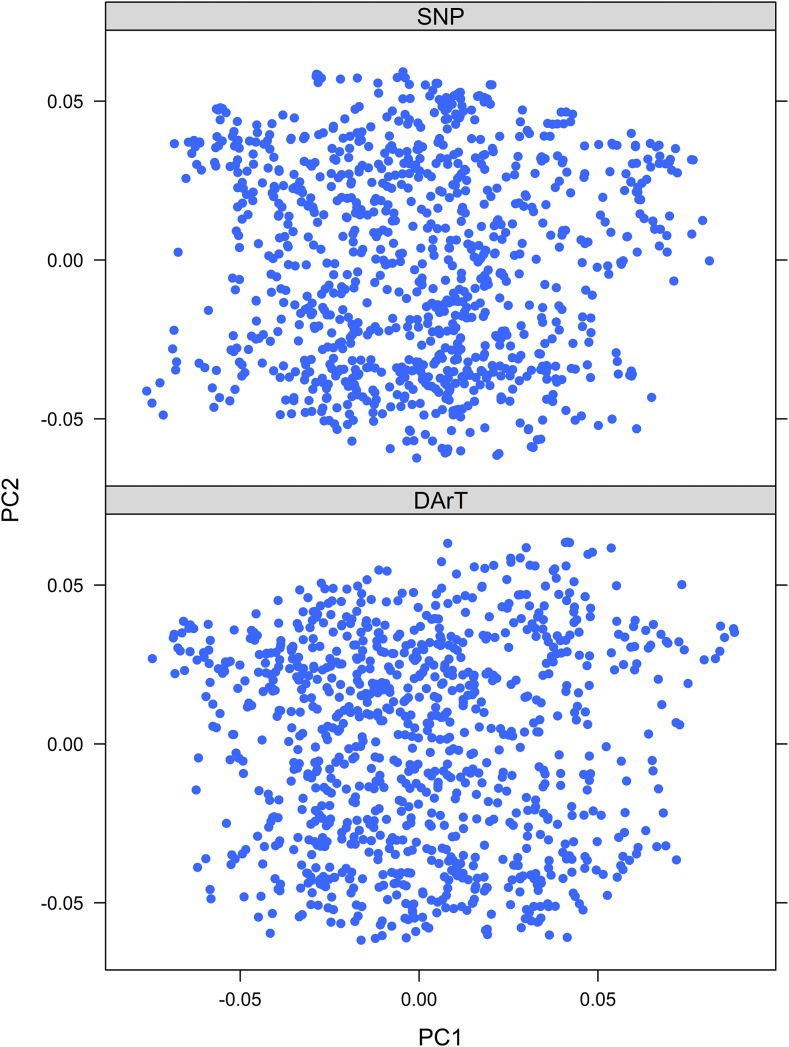
PCA plot of the first two eigenvectors from the relationship matrix of each marker set. For both marker types, the first two principle components account for ∼15% of the observed variation in genomic relationships.

Both marker sets displayed a low degree of population structure (Figure 4) with no obvious patterns among genotypes. For genomic relationship matrices computed from either SNP or DArT markers separately, the first two eigenvectors collectively explained only 15% of the variation in genomic relationships, indicating a lack of strong subpopulation structure in the association population. Only markers identified as statistically significant (with *P*-values below the 10% FDR threshold) in more than one experiment in the association studies were considered reliable associations (Table 1 and Table 2).

**Table 1:** Markers significant for water-soluble carbohydrate concentration at >1 experiment for the DArT marker set

Experiments	Marker	Chromosome	Distance (cM)	*P*-Value Range	Minor Allele Frequency	FDR Value Range	Nearby Genes
09COLE_RFD, 09YANA_RFD	*wPt-9592*	1A	68.3	0.000520–0.000789	0.491	0.0725–0.0999	
09COLE_RFD, 09YANA_RFD	*wPt-665784*	1A	69.2	0.000557–0.0006277	0.474	0.0725–0.0923	
09COLE_RFD, 09YANA_RFD	*wPt-7359*	1B	11.3	6.97 × 10^−5^ – 9.73 × 10^−5^	0.107	0.0228–0.0323	
09COLE_RFD, 09YANA_RFD	*wPt-666719*	1D	83.1	1.56 × 10^−5^ – 2.97 × 10^−5^	0.278	0.00784–0.0193	*Glu-D1*
09COLE_RFD, 09YANA_RFD	*wPt-3743*	1D	83.3	1.58 × 10^−5^ – 3.88 × 10^−5^	0.275	0.00784–0.0193	*Glu-D1*
09COLE_RFD, 09YANA_RFD	*wPt-733835*	1D	86.5	0.000205–9.16 × 10^−5^	0.285	0.0228–0.0510	*Glu-D1*
09COLE_RFD, 09YANA_RFD	*wPt-797974*	2D	41.1	0.000519–0.000607	0.378	0.0725–0.0923	*Ppd-D1*
09COLE_RFD, 09YANA_RFD	*wPt-800147*	4A	62.7	0.000804–0.000527	0.327	0.0725–0.0999	

No SNP markers showed significantly associations. Experiment is given as year-site-irrigation treatment. Chromosome and position are from the consensus map. Nearby genes are from CMAP GrainGene database (http://wheat.pw.usda.gov/cmap/) searches within the LD blocks estimated for each genome. Association analyses returned significant markers for the two water deficit environments (2009 Coleambally and Yanco Rainfed), and not any of the well-watered environment experiments.

**Table 2:** Markers significant for relative maturity at >1 experiment for the DArT and SNP marker datasets

Experiments	Marker	Chromosome	Distance (cM)	*P*-Value Range	Minor Allele Frequency	FDR Value Range	Nearby Genes
10COLE_IRR, 10COLE_RFD, 10YANA_IRR	wPt-730744	2D	73.0	4.85 × 10^−5^ – 7.91 × 10^‑5^	0.161	0.0482–0.0787	Ppd-D1
09COLE_IRR, 09COLE_RFD, 09YANA_IRR, 09YANA_RFD, 10COLE_IRR, 10COLE_RFD, 10YANA_IRR, 10YANA_RFD	wsnp_CAP12_c812_428290 (IWA989)	2D	57.9	3.91 × 10^−16^ – 1.414 × 10^−14^	0.134	1.50 × 10^−12^ – 5.41 × 10^−11^	Ppd-D1
09YANA_IRR, 09YANA_RFD	wsnp_BE422566B_Ta_1_2 (IWA76)	4B	43.3	0.000169–0.000171	0.0582	0.0498–0.0507	
09COLE_IR, 09COLE_RFD, 09YANA_IRR, 09YANA_RFD, 10COLE_IRR, 10COLE_RFD, 10YANA_IRR, 10YANA_RFD	wsnp_AJ612027A_Ta_2_1 (IWA1)	5A	66.2	6.30 × 10^−9^ – 3.74 × 10^−6^	0.360	1.20 × 10^−5^ – 0.00410	Vrn-A1
09COLE_IRR, 09COLE_RFD, 09YANA_IRR, 09YANA_RFD, 10COLE_IRR	wsnp_AJ612027A_Ta_2_5 (IWA2)	5A	66.2	1.29 × 10^−5^ – 0.000219	0.151	0.00619–0.0764	Vrn-A1
09COLE_RFD, 09YANA_IRR, 09YANA_RFD	wsnp_BE404341A_Ta_2_3 (IWA46)	5A	66.7	6.07 × 10^−5^ – 0.000105	0.136	0.0258–0.0446	Vrn-A1
09COLE_IRR, 09COLE_RFD, 09YANA_IRR, 09YANA_RFD, 10COLE_IRR, 10COLE_RFD, 10YANA_IRR, 10YANA_RFD	wsnp_BF293620A_Ta_2_1 (IWA454)	5A	66.2	1.19 × 10^−6^ – 0.000215	0.150	0.000909–0.0937	Vrn-A1
09COLE_IRR, 09COLE_RFD, 09YANA_IRR, 09YANA_RFD, 10COLE_IRR, 10COLE_RFD, 10YANA_IRR, 10YANA_RFD	wsnp_BJ224975A_Ta_2_1 (IWA589)	5A	66.2	1.53 × 10^−8^ – 4.29 × 10^−6^	0.357	1.47 × 10^−5^ – 0.00410	Vrn-A1
09COLE_RFD, 09YANA_IRR, 09YANA_RFD	wsnp_BJ224975A_Ta_2_2 (IWA590)	5A	66.2	7.20 × 10−^5^ – 0.000118	0.140	0.0276–0.0450	Vrn-A1
09COLE_IRR, 09COLE_RFD, 09YANA_IRR, 09YANA_RFD, 10COLE_IRR, 10COLE_RFD, 10YANA_IRR, 10YANA_RFD	wsnp_Ex_c22727_31934296 (IWA2743)	5A	66.7	9.26 × 10^−10^ – 6.31 × 10^−7^	0.359	1.77 × 10^−6^ – 0.00121	Vrn-A1
09COLE_RFD, 09YANA_IRR, 09YANA_RFD	wsnp_Ex_c31799_40545376 (IWA3362)	5A	69.1	0.000154–0.000243	0.369	0.0490–0.0581	Vrn-A1
09COLE_IRR, 09COLE_RFD, 09YANA_IRR, 09YANA_RFD, 10COLE_IRR, 10COLE_RFD	wsnp_Ex_c31799_40545478 (IWA3363)	5A	69.1	1.74 × 10^−6^ – 0.000195	0.379	0.0009498–0.0747	Vrn-A1
09COLE_IRR, 09COLE_RFD, 09YANA_IRR, 09YANA_RFD, 10COLE_IRR, 10COLE_RFD	wsnp_Ex_c7729_13177883 (IWA4719)	5A	69.1	1.43 × 10^−6^ – 0.000152	0.379	0.000911–0.0647	Vrn-A1
09COLE_RFD, 09YANA_IRR, 09YANA_RFD	wsnp_Ex_rep_c66689_65010988 (IWA5287)	5A	66.7	0.000126–0.000203	0.400	0.0438–0.0518	Vrn-A1
09YANA_IRR, 09YANA_RFD, 10COLE_IRR, 10COLE_RFD, 10YANA_IRR, 10YANA_RFD	wsnp_Ex_c12048_19288999 (IWA1577)	5B	71.1	3.83 × 10^−5^ – 0.000223	0.266	0.0291–0.0533	Vrn-B1
09YANA_IRR, 09YANA_RFD	wsnp_Ra_c20970_30293078 (IWA7732)	5B	71.1	0.000332–0.000433	0.272	0.0747–0.0976	Vrn-B1
10YANA_IRR, 10YANA_RFD	wsnp_Ra_c20970_30293227 (IWA7733)	5B	71.1	6.16 × 10^−5^ – 0.000183	0.262	0.0383–0.0499	Vrn-B1
10YANA_IRR, 10YANA_RFD	wsnp_Ex_c508_1008029 (IWA4087)	5D	61.0	3.90 × 10^−5^ – 0.000332	0.202	0.0258–0.0978	Vrn-D1

Experiment is given as year-site-irrigation treatment. Chromosome and position are from the consensus maps. Nearby genes are from CMAP GrainGene database (http://wheat.pw.usda.gov/cmap/) searches within the LD blocks estimated for each genome.

The GWAS for relative maturity using the DArT markers identified only one marker (on chromosome 2D), which was detected as significant in three experiments, all of them in the well-watered environment cluster (10COLE_IRR, 10COLE_RFD, and 10YANA_IRR; Table 2). This marker is located >30 cM from the marker associated with WSCC on the same chromosome. In contrast, GWAS for relative maturity using the SNP marker set identified 17 markers significant in more than one experiment (Table 2). SNP associations with relative maturity within specific experiments did not follow the pattern of environmental clustering observed for WSCC. Rather, five of 17 SNP associations with relative maturity were observed in all experiments. Some environmental-specificity was observed for relative maturity associations, but this did not reflect differences between well-watered and water-limited conditions. For example, four of 17 SNP associations were detected at both treatments within the same year-location combination (Table 2), suggesting G × E patterns for relative maturity due to local weather patterns rather than water availability.

Among the SNPs associated with relative maturity, 11 markers were located within 3 cM of each other on the consensus map on chromosome 5A, and three markers collocated on chromosome 5B. Additionally, one marker was identified on each of chromosomes 2D, 4B, and 5D. Four of the significant markers on 5A and the marker on 2D were detected in all eight experiments.

The range in MAF of trait-associated loci ranged from 0.107 to 0.491 for the DArT marker set, and at least one relatively rare SNP allele was detected for relative maturity (MAF = 0.058 on chromosome 4B). The highest MAF for associated loci in the SNP marker set was 0.400.

## Discussion

### Comparison of analysis at individual experiments

GWAS results for both relative maturity and WSCC show that significant associations can be experiment-specific, and relatable to the overall G × E relationships between experiments for each trait. Genetic correlations between all experiments were very high for relative maturity (indicating limited G × E interaction), and significant loci were detected in all experiments. In contrast, G × E was strong for WSCC, with factor analysis revealing two distinct environment types, corresponding to well-watered and water-deficit environments. Reflecting these differences in overall G × E patterns between relative maturity and WSCC, several markers were associated with relative maturity across all experiments, whereas the significant associations for WSCC were detected only in two experiments in the water deficit environment cluster. No significant associations for WSCC were detected in the well-watered environment cluster of experiments.

Combinations of well-watered and water deficit environments have been used for WSCC QTL detection previously, and, in some studies, such as Yang *et al.* (2007) and Pinto *et al.* (2010), QTL for WSCC were detected in well-watered, or water deficit environments but not in both. This discrepancy illustrates the importance of environmental characterization in QTL analysis, and the value in understanding the target population of environments that each QTL analysis is performed in. Once established, GWAS can be conducted separately for each experiment, or separately for traits values averaged over environments within well-defined clusters. Experiment-by-experiment GWAS should allow a means to replicate QTL detection in comparable environments, and as both Oldmeadow *et al.* (2011) and Zila *et al.* (2013) indicate, to understand possible QTL × environment interactions.

### Loci associated with WSCC

GWAS detected associations between markers on chromosomes 1A, 1B, 1D, 2D, and 4A with WSCC measured in water-limited conditions. No markers were associated with both WSCC and relative maturity, in contrast to Rebetzke *et al.* (2008), where flowering time loci explained large proportions of variation for WSCC. The results herein may reflect the sampling methods used for phenotyping WSCC, or because the association population lines for WSCC were selected to be constrained for development.

Among the markers significantly associated with WSCC in the two water deficit environment experiments, *wPt-7359* on 1B has not been previously reported in trait associations, but *wPt-800147* on 4A was associated with plant height (Yu *et al.* 2014) and seedling shoot dry weight under normal and saline conditions (Masoudi *et al.* 2015). Marker *wPt-3743* on 1D was associated with a range of other traits, including grain yield and resistance to yellow rust, powdery mildew, and leaf rust (Crossa *et al.* 2007), grain yield and spike length under salt stress conditions (Azadi *et al.* 2015), and spike number (tiller number) per plant (Cui *et al.* 2014). Marker *wPt-9592* on 1A was previously associated with grain yield under water deficit conditions (in particular, terminal drought; Crossa *et al.* 2007), heading date after vernalization (Le Gouis *et al.* 2012), and seed dormancy (Singh *et al.* (2010).

Marker *wPt-3743* on chromosome 1D was previously reported to be located near the high molecular weight glutenin *Glu-D1* locus and the storage protein activator gene locus *SPA-D* (Plessis *et al.* 2013; Deng *et al.* 2015; Jin *et al.* 2015). Marker *wPt-733835* is also in this region (Jin *et al.* 2015). The *Glu-D1* locus is important for selection as, along with the *Glu-A1* and *Glu-B1* loci, it is responsible for a large percentage of the phenotypic variation for dough quality. The combination of glutenin alleles present at the *Glu-1D* locus will largely determine the end use and grain quality class of wheat varieties (Payne 1987; Whiting 2004). Glutenin protein complexes play an important role in conferring elasticity and strength in wheat dough (Plessis *et al.* 2013), and the *Glu* loci have been shown to collocate with QTL for nitrogen and dry matter accumulation in grain (Charmet *et al.* 2005).

Rebetzke *et al.* (2008) identified QTL for WSC per tiller that collocated with the glutenin loci *Glu-A1*, and *Glu-B1*. The *Glu-D1* and *SPA-D* loci contribute to phenotypic variation for grain yield and grain number through the plant response to nitrogen (Bordes *et al.* 2013). Potentially the *Glu* loci could be involved with the inheritance of WSCC through an interaction between nitrogen use, tiller number, and grain weight. WSCC is influenced by nitrogen content, as higher nitrogen availability in the plant drives sink demand for assimilate (van Herwaarden *et al.* 1998; Ruuska *et al.* 2008), and WSC tends to accumulate in the absence of sink demand (Gebbing 2003).

### Loci associated with relative maturity

Significant associations were identified in the vicinity of a number of the known major flowering time loci, including the main photoperiod and vernalization loci under selection in wheat breeding germplasm pool globally (Yan *et al.* 2004; Eagles *et al.* 2009, 2010; Cane *et al.* 2013; Slafer *et al.* 2015). Both DArT and SNP markers were identified close to the photoperiod-sensitivity locus *Ppd-D1* on chromosome 2D on the consensus map (Table 1). Given the importance of the *Ppd-D1* locus to selection of growth duration and adaptability (Kamran *et al.* 2014; Slafer *et al.* 2015), the SNP and DArT markers identified here may prove useful to supplement other markers for this locus, such as those outlined in Cane *et al.* (2013).

The analyses were able to detect significant associations near the *Vrn-A1*, *Vrn-B1*, and *Vrn-D1* loci across multiple experiments, although only SNP markers were identified as statistically significant, including 11 markers near *Vrn-A1*, three markers near *Vrn-B1*, and one marker was detected near *Vrn-D1* (Table 1). For the genotypes in this study, variation in at these loci would be expected to include alleles for both spring and winter alleles, as well as winter alleles that confer different vernalization requirements (Eagles *et al.* 2010, 2014; Harris *et al.* 2017). One marker identified (*wsnp_AJ612027A_Ta_2_1*) was also reported to be associated with the *Vrn-A1* locus by Lopes *et al.* (2015). Three markers identified on 5B are within 10 cM of the *Vrn-B1* locus reported by Guedira *et al.* (2014). The single marker on chromosome 5D associated with relative maturity (*wsnp_Ex_c508_1008029*) at both 10YANA_IRR and 10YANA_experiments corresponds to the vicinity of the *Vrn-D1* locus (Eagles *et al.* 2009).

An additional marker associated with relative maturity at both the 09YANA_IRR and 09YANA_RFD experiments was located on chromosome 4B (*wsnp_BE422566B_Ta_1_2*). This marker was significant in both the water deficit environments as well as the well-watered environments in this study. QTL for heading date on this chromosome have been previously reported by Hanocq *et al.* (2007), Griffiths *et al.* (2009), and Le Gouis *et al.* (2012), and photoperiod sensitivity QTL on this chromosome have been reported by Shindo *et al.* (2003) and Sourdille *et al.* (2003).

### Comparison of DArT and SNP molecular marker sets

DArT markers were developed using methylation-sensitive restriction enzymes, and, as such, can represent methylation polymorphisms that provide both genetic and epigenetic information (Wenzl *et al.* 2004; Akbari *et al.* 2006). It is also possible for DArT markers to be located in insertion/deletion sites (indels), although ∼80% are SNPs (Kilian *et al.* 2005). The minor allele frequency distribution for both marker sets was close to uniform across most of the allele frequency range, in contrast to the expected inflation of rare alleles expected for markers under drift–mutation equilibrium (Hamblin *et al.* 2011). This frequency spectrum in the SNP marker set was noted previously by Cavanagh *et al.* (2013), who concluded “The observed MAF is the consequence of intentional bias in SNP selection, where common alleles were favored by choosing more broadly distributed SNPs.”

Our genotype set had more than twice as many polymorphic SNP markers than DArT markers, and the DArT marker set had a higher proportion of missing data. Across the A and B genomes, DArT markers exhibit a more rapid breakdown of LD than SNP markers, but the pattern was reversed in the D genome. The longer linkage blocks in the D genome for both SNP and DArT markers is consistent with previous reports (Wang *et al.* 2014). The differences in marker density, distribution and LD structure between the two markers sets are the most likely causes of the observed differences in association analyses.

### Conclusions

This study highlights the need to characterize G × E interactions in multi-environment datasets, and to define target populations of environments for marker-trait associations. These populations of environments define the scope of inference for interpreting GWAS results. In this study, we identified two clusters of experiments based on their genotypic correlations for the expression of WSCC. Marker associations for WSCC were identified only in the water deficit experiments, which represented a minority of the experiments; these associations would have been missed if the trait values were averaged across all experiments.

The loci identified for WSCC have both previously been associated with performance under water limited conditions, but did not reflect linkage to major effect relative maturity loci. The marker on 1D colocates with the *Glu-D1* locus, which may have some pleiotropic effect on WSCC. These reported associations may be useful for marker-assisted selection of WSCC in water-limited environments, independent of relative maturity.

## Supplementary Material

Supplemental material is available online at www.g3journal.org/lookup/suppl/doi:10.1534/g3.117.039842/-/DC1.
