# Supplementary material for: Genome-Wide Associations for Water-Soluble Carbohydrate Concentration and Relative Maturity in Wheat Using SNP and DArT Marker Arrays
Source: G3 (Bethesda). 2017 Jun 27;7(8):2821–30. doi: 10.1534/g3.117.039842 (PMC5555485; doi:10.1534/g3.117.039842)
Supplement: Supplementary file 1 [file 2821FileS1.docx]

## Supplemental data files

File S2 contains phenotype information for WSCC including both field and laboratory phase experimental blocking factors. File S3 contains phenotype information for relative maturity including field experimental blocking factors.

File S4 contains SNP genotypes for each individual. File S5 contains DArT genotypes for each individual. Both of these genotype files have been modified to approximate the HAPMAP format required for GAPIT analyses, where the AA/BB format of the 9K SNP data and the 0/1 format of DArT data has been substituted with AA/CC. Position relates to the consensus map position in centimorgans. Chromosomes are numbered 1-21 corresponding to the following table:

| Chromosome | Chromosome number |
| --- | --- |
| 1A | 1 |
| 1B | 2 |
| 1D | 3 |
| 2A | 4 |
| 2B | 5 |
| 2D | 6 |
| 3A | 7 |
| 3B | 8 |
| 3D | 9 |
| 4A | 10 |
| 4B | 11 |
| 4D | 12 |
| 5A | 13 |
| 5B | 14 |
| 5D | 15 |
| 6A | 16 |
| 6B | 17 |
| 6D | 18 |
| 7A | 19 |
| 7B | 20 |
| 7D | 21 |
